# 12-Hydroxyjasmonic acid glucoside causes leaf-folding of *Samanea saman* through ROS accumulation

**DOI:** 10.1038/s41598-022-11414-2

**Published:** 2022-05-04

**Authors:** Gangqiang Yang, Yasuhiro Ishimaru, Shunji Hoshino, Yuki Muraoka, Nobuyuki Uozumi, Minoru Ueda

**Affiliations:** 1 Department of Chemistry, Graduate School of Science, Tohoku University, Sendai, 980-8578 Japan; 2 Department of Mechanism and Chemical Life Sciences, Graduate School of Life Sciences, Tohoku University, Sendai, 980-8578 Japan; 3 Graduate School of Engineering, Tohoku University, 6-6-07 Aoba-ku, Aobayama, Sendai, 980-8579 Japan; 4 Present Address: School of Pharmacy, Collaborative Innovation Center of Advanced Drug Delivery System and Biotech Drugs in Universities of Shandong, Key Laboratory of Molecular Pharmacology and Drug Evaluation, Ministry of Education, Yantai University, Yantai, 264005 China

**Keywords:** Chemical biology, Jasmonic acid

## Abstract

Foliar nyctinasty, a circadian rhythmic movement in plants, is common among leguminous plants and has been widely studied. Biological studies on nyctinasty have been conducted using *Samanea saman* as a model plant. It has been shown that the circadian rhythmic potassium flux from/into motor cells triggers cell shrinking/swelling to cause nyctinastic leaf-folding/opening movement in *S. saman*. Recently, 12-hydroxyjasmonic acid glucoside (JAG) was identified as an endogenous chemical factor causing leaf-folding of *S. saman*. Additionally, SPORK2 was identified as an outward-rectifying potassium channel that causes leaf-movement in the same plant. However, the molecular mechanism linking JAG and SPORK2 remains elusive. Here, we report that JAG induces leaf-folding through accumulation of reactive oxygen species in the extensor motor cells of *S. saman*, and this occurs independently of plant hormone signaling. Furthermore, we show that SPORK2 is indispensable for the JAG-triggered shrinkage of the motor cell. This is the first report on JAG, which is believed to be an inactivated/storage derivative of JA, acting as a bioactive metabolite in plant.

## Introduction

Circadian rhythmic leaf-folding, called nyctinasty, is a widely observed physiological behavior of leguminous plants^1,2^, wherein the plants open their leaves in the morning and fold them in the evening. The rhythm of this phenomenon is not affected by environmental conditions and occurs even under continuous light/dark conditions. The pulvinus, a specialized organ located in the base of legume leaflets, bends and straightens according to the circadian rhythm^3^. Unequal volume changes in the motor cells in the adaxial/abaxial side of the pulvinus cause the leaf movement.

**Figure 1:**
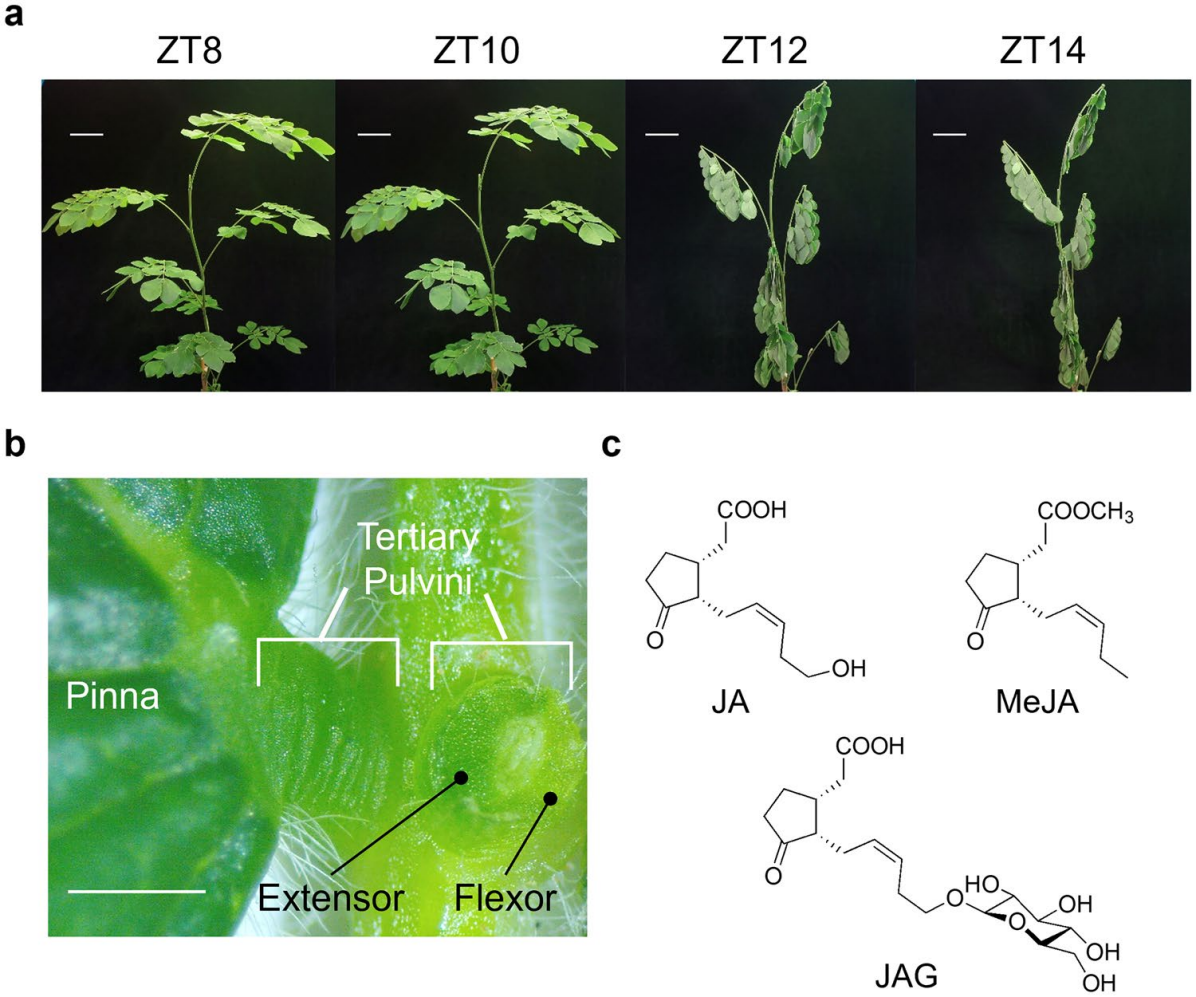
*Samanea saman* is a standard plant in the study of nyctinasty. (**a**) Images of *S. saman* during a quarter of circadian rhythm from ZT 8–14 showing leaf-folding movement. Bar = 10 cm. (**b**) Enlarged image of the tertiary pulvini of *S. saman*. Note that adaxial side is extensor and abaxial side is flexor. Bar = 1 mm. (**c**) Chemical structures of jasmonates: jasmonic acid (JA), methyl jasmonate (MeJA), and 12-hydroxyjasmonic acid glucoside (JAG). Note that JAG is the endogenous chemical factor that induces leaf-folding in *S. saman*.

The earliest record of nyctinasty dates back to the reign of Alexander the Great in 400 B.C^4^. Later, in the eighteenth century, the first discovery of a biological clock was reported in the context of the nyctinastic leaf-movement of *Mimosa pudica*^5^. In his later years, Charles Darwin devoted himself to the study of plants, and at the end of the nineteenth century published a paper entitled “The Power of Movement in Plants,” wherein he summarized his extensive observations of plant movement^6^. *Samanea saman* was established as a standard plant for the study of nyctinasty in 1958^7,8^. However, focused research on the physiological basis for plant nyctinasty was not conducted until the 1970–1990s^9^. During this period, a number of landmark studies on *Samanea saman* (Fig. 1) were reported, such as those on potassium flux and leaf movement^10,11^, relationship between leaf movement and the biological clock^12,13^, the identification of motor cell as the primary cause of leaf-movements^14,15^, electrophysiological studies on putative ion channels^16–19^ and aquaporin^20^, and the effects of red/blue light^21,22^.

Previously, we reported that leaf-movements in leguminous plants are controlled by endogenous chemical factors^23–25^. We focused our attention on 12-*O*-β-D-glucopyranosyl-jasmonic acid, also referred to as jasmonic acid glucoside (JAG, or also called LCF in ref 21), a leaf-folding substance in *S. saman* (Fig. 1)^26^, and found that JAG binds to extensor motor cells located in the pulvinus on the adaxial side of the leaf through the putative target membrane target protein of jasmonate glucoside (MTJG)^27^. JAG was found to induce shrinking of the protoplast prepared from *Samanea* adaxial motor cells (extensor cells) but did not affect protoplasts from the abaxial motor cells (flexor cells)^26^. It is possible that JAG-induced shrinking occurs either in a COI1-independent or COI1-dependent manner because *Samanea* has a modified COI1-JAZ that also binds to JAG. In addition, we identified SPORK2, a potassium channel responsible for the leaf-movement of *S. saman*^28,29^. However, the mechanistic basis for the induction of K^+^ release via activation of the potassium channel by JAG remains elusive. Here, we report that JAG induces the accumulation of reactive oxygen species (ROS) in the adaxial motor cells of *S. saman* to induce cell shrinking. JAG-induced shrinking of adaxial cells occurs independently of the plant hormones jasmonic acid (JA) and abscisic acid (ABA), which are also known to induce shrinking of guard cells. This finding will pave the way to a complete understanding of the molecular mechanism of JAG-triggered nyctinastic leaf-closing in *S. saman*. 

## Results

### JAG-induced accumulation of second messengers in motor cells

The shrinking of stomatal guard cells during stomatal closure is well known in the field of cell-shrinking in plants, and a plethora of molecules involved in ABA-induced stomatal cell shrinking have been identified^30,31^. In particular, ROS and calcium ions are known to play an important role as second messengers^32,33^. ROS are effective signaling molecules that can induce guard cell shrinkage in both methyl jasmonate (MeJA)- and ABA-induced stomatal closure^34^. We compared the effect of JAG with that of plant hormones ABA and JA both in *Samanea* extensor motor cells and *Arabidopsis* guard cells. It is also possible that 12-OH-JA, the hydrolyzed product and a biosynthetic precursor of JAG, might function as the bioactive form of JAG. However, in our previous report, we confirmed that 12-OH-JA did not induce shrinkage of *Samanea* motor cells^26^; hence, 12-OH-JA was excluded from the experiments in this study.

**Figure 2:**
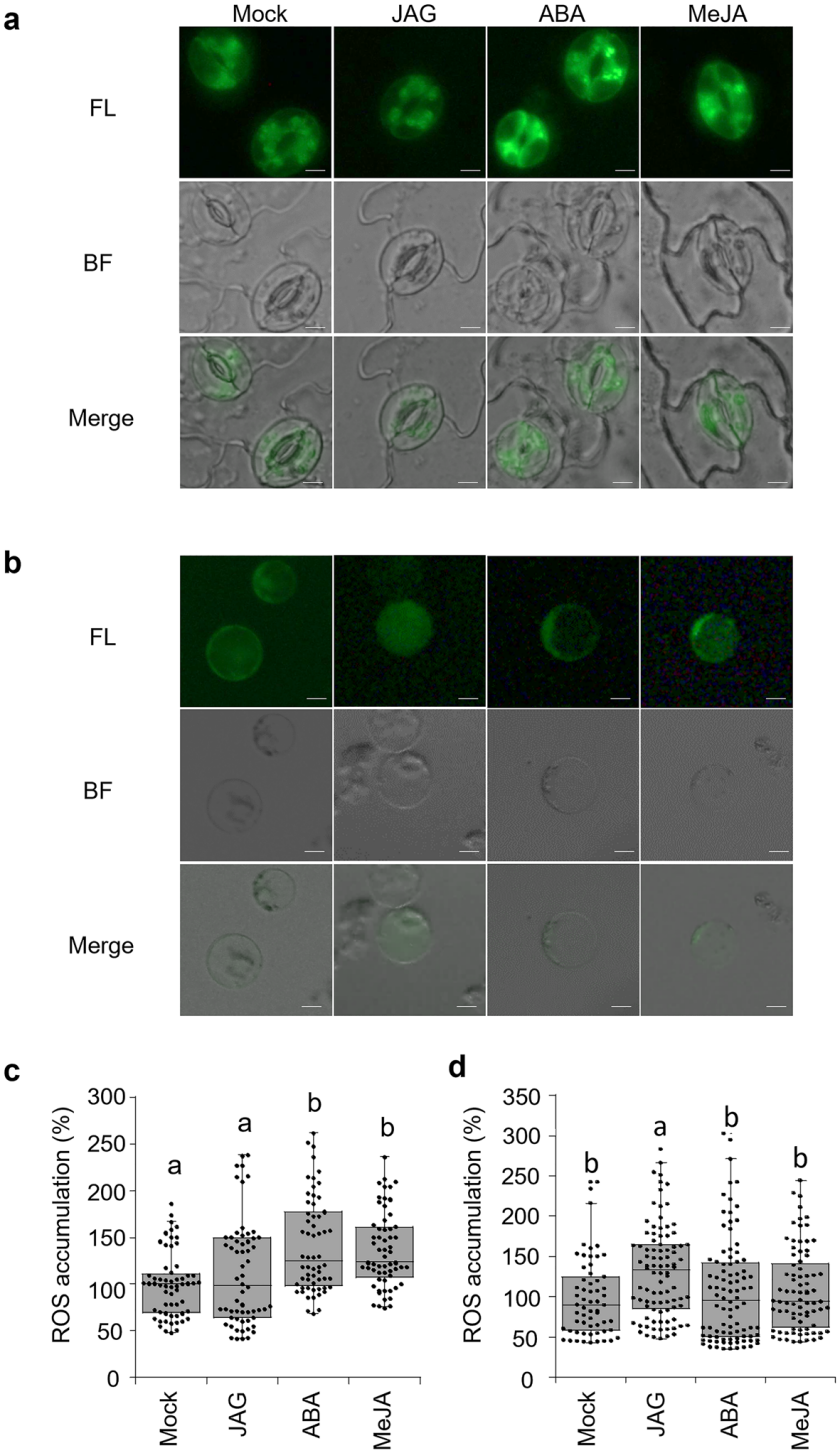
JAG induces ROS accumulation in tertiary extensor motor cell protoplasts of *Samanea saman.* (**a**, **b**) Representative images of reactive oxygen species (ROS) accumulation visualized by fluorescent dye DCF. (**a**) Epidermal peels of *Arabidopsis thaliana* or (**b**) tertiary extensor motor cell protoplasts of *S. saman* were loaded with H_2_DCFDA for 30 min before adding 100 µM 12-hydroxyjasmonic acid glucoside (JAG), 10 µM abscisic acid (ABA), or 10 µM methyl jasmonate (MeJA). Photomicrographs were taken 20 min or 15 min after treatment (*A. thaliana*) or at 15 min after treatment (*S. saman*). The experiments using *S. saman* protoplasts were performed during ZT 6–11. Bar = 10 µm. (**c**) Quantification of ROS accumulation in guard cells of *A. thaliana* following above treatment as in (**a**). Box plot represents fluorescence intensity ratio of treated cells to mock cells. All data are represented as dot plots (n = 60). Mean ± SE are as follows: Mock, 100.0 ± 4.5; JAG, 141.5 ± 6.5; ABA, 107.4 ± 3.9; MeJA, 111.9 ± 7.3. (**d**) Quantification of ROS accumulation in tertiary extensor motor cell protoplasts of *S. saman* following above treatment as in (**b**). Box plot represents fluorescence intensity ratio of treated cells to mock cells_._ All data are represented as dot plots (n = 61–92). Mean ± SE are as follows: Mock, 100.0 ± 6.4; JAG, 131.5 ± 5.9; ABA, 106.3 ± 6.7; MeJA, 108.4 ± 5.6. Different letters indicate significant differences (SNK post-hoc test, *P* < 0.05) in (**c**) and (**d**).

**Figure 3:**
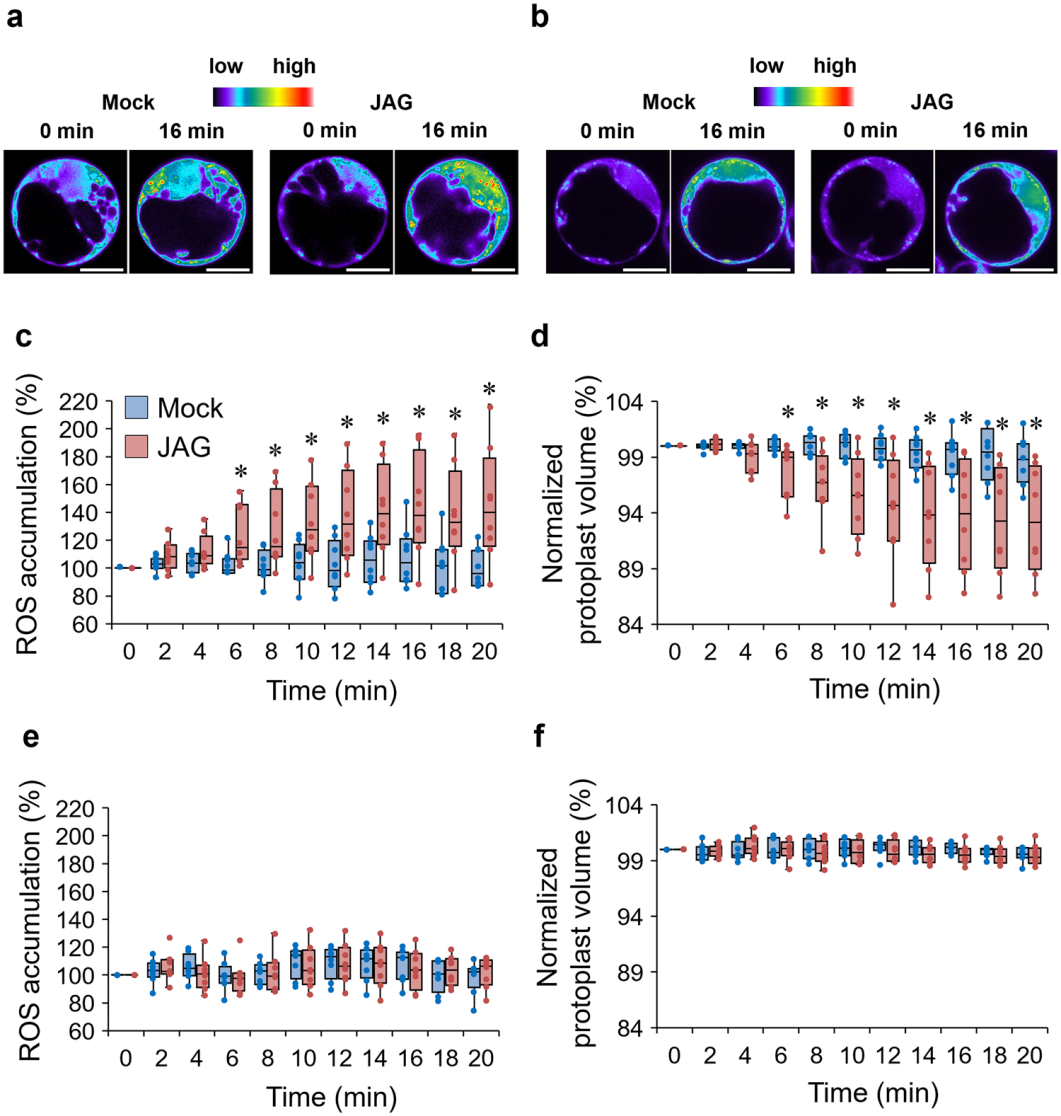
JAG-induced ROS accumulation is involved in leaf-close movement in *Samanea saman*. (**a**, **b**) Representative images of reactive oxygen species (ROS) accumulation in (**a**) extensor or (**b**) flexor indicated by fluorescent dye DCF. Motor cell protoplasts of *S. saman* were loaded with H_2_DCFDA for 45 min before adding 100 µM 12-hydroxyjasmonic acid glucoside (JAG). Photomicrographs were taken using a confocal laser scanning microscope at 0 and 16 min after above treatments. Bar = 10 µm. (**c**, **d**) Box plots represent time course of JAG-induced (**c**) cytosolic ROS accumulation and (**d**) cell shrinkage in tertiary extensor protoplasts of *S. saman* following above treatment as in (a). All data points are shown as dot plots (n = 8). (**e**, **f**) Box plots represent time course of JAG-induced (**e**) cytosolic ROS accumulation and (**f**) cell shrinkage in tertiary flexor protoplasts of *S. saman* following above treatment as in (**b**). All data points are shown as dot plots (n = 8). Asterisks indicate significant differences (*p < 0.05; *t*-test) in (**c**)–(**f**). All experiments were conducted during ZT 6–11. Four independent experiments were performed.

Protoplasts of *Arabidopsis* guard cells and *Samanea* motor cells were prepared as previously reported^15^. Intracellular ROS accumulation in these protoplasts was monitored using the fluorescent dye 2′, 7′-dichlorofluorescin diacetate (H_2_DCF-DA), which is widely used as a ROS indicator in guard cells^35,36^. Significant ROS accumulation (*p* < 0.05 by SNK post-hoc test) was triggered in the guard cells of *A. thaliana* by 10 µM ABA treatment (Fig. 2a), which is consistent with previous reports^35,36^. No ROS accumulation was observed in guard cells of *A. thaliana* treated with 100 µM JAG (Fig. 2a and c). In contrast, significant ROS accumulation (*p* < 0.05 by SNK post-hoc test) was triggered in protoplasts isolated from *Samanea* extensor motor cells by 100 µM JAG, whereas 100 µM ABA had no distinct effect on ROS generation in motor cells (Fig. 2b and d). In addition, JAG-induced ROS accumulation was not observed in *Samanea* flexor motor cells, wherein JAG cannot induce cell shrinking (Fig. 3b and f)^26^. It was concluded that JAG triggered ROS accumulation in extensor motor cell protoplasts, whereas ABA triggered ROS accumulation in guard cells, and that the responses to JAG and ABA in the *Arabidopsis* and *Samanea* protoplasts were very different. This result suggests that the modes of actions of JAG and ABA are different, and that responses to endogenous chemicals in *Arabidopsis* guard cells and *Samanea* motor cells are different.

MeJA is widely used to trigger JA signaling in plants^37^. Like ABA, MeJA also induces stomatal closure^34,38^. However, JA cannot induce shrinkage of extensor motor cells^26^. In our study, treatment with 10 µM MeJA triggered ROS accumulation in *Arabidopsis* guard cells (Fig. 2a and c), a result consistent with previous reports^35,36^. In extensor motor cell protoplasts, JAG treatment triggered ROS accumulation, whereas treatment with 100 µM MeJA did not affect the ROS accumulation within 15 min (Fig. 2b and d). These results are consistent with the previous finding that JAG is an inactivated derivative of JA and cannot induce JA signaling^39^. These results suggested that JAG-triggered ROS accumulation is independent of JA signaling. Thus, we further focused on the JAG-induced accumulation of ROS in the *Samanea* motor cells.

### JAG-induced ROS accumulation in the cytosol triggers shrinkage of extensor motor cell protoplasts

Recent studies have shown that ROS accumulates in different subcellular components by different mechanisms^40^. In general, chloroplasts are the largest ROS producers in plants, especially in periods of light. In contrast, mitochondria produce most ROS in the dark and in non-green tissues^40^. JAG treatment triggered ROS accumulation in the extensor motor cell protoplasts of *S. saman*. Understanding the subcellular distribution of the accumulated ROS will provide insight into its mechanisms of action.

We analyzed the subcellular distribution of JAG-induced ROS in extensor motor cell protoplasts using double staining experiments with H_2_DCF-DA and MitoTracker Red CMXRos. Chloroplasts were identified by autofluorescence. The background DCF signal in the Mock treatment strongly colocalized with chloroplasts and mitochondria (Fig. S1). The JAG treatment caused a remarkable increase in the DCF signal in the cytosol (Fig. S1), demonstrating that JAG-triggered ROS accumulates in the cytosol.

Next, we examined whether JAG simultaneously induced ROS accumulation and cell shrinkage in *Samanea* motor cell protoplasts isolated from *Samanea* extensor/flexor motor cells prepared from the adaxial/abaxial side of the pulvinus, respectively (Fig. 3). Although JAG induced ROS accumulation was observed in the cytosol of extensor motor cell protoplasts, there was no observable ROS accumulation in the cytosol of flexor motor cell protoplasts (Fig. 3a, b and c, e). Significant cell shrinkage (*p* < 0.05 by *t*-test) was observed when extensor motor cell protoplasts but not flexor motor cell protoplasts were treated with JAG (Fig. 3d and f), consistent with a previous result^26^. The time-dependent change in JAG-triggered cytosolic ROS accumulation was consistent with that of JAG-triggered shrinkage of extensor motor cell protoplasts (Fig. 3c and d). The extensor motor cell protoplasts started to shrink within several minutes and reached a plateau within 15 min of JAG treatment, and the JAG-triggered cytosolic ROS accumulation followed the same time-course. In contrast, JAG treatment had no effect on either ROS accumulation or cell shrinkage of flexor motor cell protoplasts (Fig. 3e and f). Together, these results suggest that JAG-triggered ROS accumulation may be involved in JAG-induced extensor motor cell shrinkage.

To further assess JAG-induced ROS accumulation, a series of solutions of varying H_2_O_2_ concentration was applied to the protoplasts instead of JAG. Within 20 min, both 100 µM and 1000 µM H_2_O_2_ significantly increased ROS accumulation (*p* < 0.01 by *t*-test) in the cytosol of extensor motor cell protoplasts by up to 40 and 400%, respectively, whereas no increase in ROS accumulation was observed in protoplasts treated with 10 µM H_2_O_2_ (Fig. S2). The effect of 100 µM H_2_O_2_ on ROS accumulation in the cytosol of extensor motor cell protoplasts was similar to that of JAG (Figs. 3c and S2). Therefore, 100 µM H_2_O_2_ was used in the subsequent cell volume change experiments. The effect of JAG on the cytosolic ROS accumulation in extensor motor cell protoplasts could be replicated using 100 µM H_2_O_2_, which could also induce the shrinkage of extensor motor cell protoplasts (Fig. S3). This result suggests that JAG induces extensor motor cell shrinkage through ROS accumulation.

**Figure 4:**
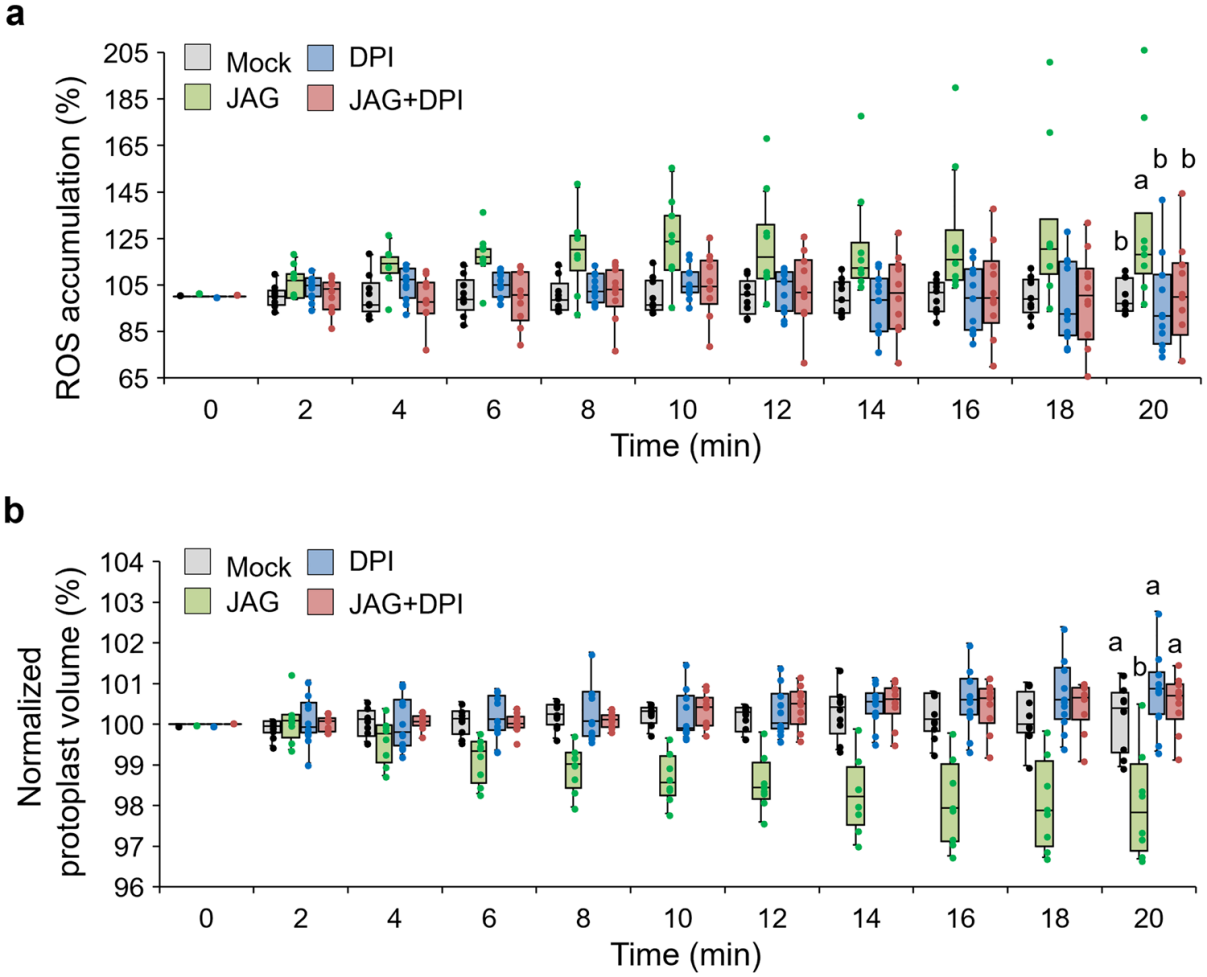
Effect of DPI on JAG-induced ROS accumulation in tertiary extensor of *Samanea saman*. (**a**, **b**) Effects of diphenyleneiodonium chloride (DPI) on 12-hydroxyjasmonic acid glucoside (JAG)-induced (**a**) cytosolic reactive oxygen species (ROS) accumulation and (**b**) cell shrinkage in tertiary extensor protoplasts of *S. saman*. Protoplasts were preincubated for 30 min with 12.5 µM DPI before adding 100 µM JAG. All data points are shown as dot plots (n = 8–11). Different letters indicate significant differences (SNK post-hoc test, *P* < 0.05). All experiments were conducted during ZT 6–11. Four independent experiments were performed.

To further assess the role of JAG-induced ROS accumulation in JAG-induced cell shrinkage, the effects of exogenous diphenyleneiodonium chloride (DPI), a widely used inhibitor of ROS production by flavoproteins including RbOH^34,41,42^, and catalase, an H_2_O_2_ scavenger^43–45^, were investigated. Cytosolic ROS accumulation and JAG-induced cell shrinkage were both restricted, even after JAG treatment, in the presence of 12.5 µM DPI (Fig. 4). Furthermore, the exogenous application of 100 units mL^-1^ catalase prior to JAG treatment strongly repressed cytosolic ROS accumulation and eliminated JAG-induced cell shrinkage (Fig. S4). These results emphasize the importance of ROS accumulation in JAG-triggered extensor motor cell shrinkage.

### Expression of outward-rectifying K^+^ channels is indispensable for JAG-induced shrinkage of extensor motor cell protoplasts

The involvement of K^+^ efflux systems in the JAG-induced shrinkage of *Samanea* extensor motor cell protoplasts was previously reported^16,17^. Recently, we revealed that *SPORK2*, a gene encoding the outward-rectifying K^+^ channel of *S. saman*, was responsible for leaf-opening movement^28^. Accordingly, we further examined the role of *SPORK2* in JAG-triggered cell shrinkage. Unfortunately, we could not observe direct activation of SPORK2 by the addition of JAG (data not shown). However, we did observe the ion transport activity of SPORK2 in whole-cell patch clamp experiment using isolated *Samanea* motor cells, as previously reported^28^. In these experiments, signal transduction from NADPH oxidase to SPORK2 did not occur because the cytosolic contents of *Samanea* motor cells were replaced by the pipette solution. However, when treated with H_2_O_2_, SPORK2 expressed in *Xenopus* oocyte was activated (Fig. S5).

Next, we examined the effect of JAG on extensor motor cells when the expression of *SPORK2* was the lowest. To obtain extensor motor cells with the lowest expression level of *SPORK2*, we checked the expression of *SPORK2* in a quarter period of diurnal rhythm containing leaf-folding movement (Fig. 1). *SPORK2* expression was highest at Zeitgeber time (ZT) 8 and decreased as the leaves gradually folded, and the *SPORK2* expression was not fluctuant in the flexor during this quarter. In the extensors, expression of *SPORK2* reached a nadir at ZT 14. In addition, immunostaining using anti-SPORK2 antibody demonstrated that SPORK2 in tertiary pulvinus decreased as *SPORK2* gene expression decreased (Fig. S6). We also examined the effect of JAG on the extensor motor cell protoplasts after ZT 14. Interestingly, significant ROS accumulationwas observed in extensor motor cell protoplasts under JAG treatment, whereas no cell shrinkage was observed. Thus, JAG cannot cause shrinkage of extensor motor cell protoplasts with the lowest expression level of *SPORK2* gene. Meanwhile, in flexor motor cells, there was no specific ZT that caused JAG-induced ROS accumulation as well as cell shrinkage (Fig. S7).

## Discussion

*Samanea saman* is a model plant used in legume nyctinastic leaf movement studies. Recently, JAG was identified as the bioactive metabolite that mediates this leaf-folding movement in *S. saman*^25,46^. JAG is perceived by the extensor motor cells in the *Samanea* plant body^27^, and it selectively induces extensor motor cell shrinkage to cause leaf closure^26^. This is the first report on the molecular mechanism of JAG, which is believed to be a deactivated/storage derivative of JA^39,47^, as a bioactive metabolite in plant.

This study examined the involvement of ROS accumulation in the JAG-induced motor cell shrinkage of *S. saman*. The fluorescent dye H_2_DCF-DA was used to detect ROS accumulation. ABA and MeJA triggered ROS production in the guard cells of *A. thaliana*, as reported previously^34–36^, whereas JAG was found to trigger ROS accumulation in motor cell protoplasts of *S. saman*. This result demonstrated that JAG-induced ROS accumulation is independent of ABA/JA signaling. The distribution of ROS accumulation was visualized by confocal laser scanning microscopy (CLSM). JAG-triggered ROS accumulation and JAG-induced cell shrinkage were simultaneously examined in the extensor/flexor motor cells of the *Samanea* pulvinus. The effect of exogenous DPI chloride (a widely used inhibitor of RbOHs)^34^, catalase (a ROS scavenger)^43,48^ and H_2_O_2_ were all examined. Finally, the mRNA expression of the main outward-rectifying K^+^ channel (SPORK2) was detected, as well as its effect on JAG-triggered ROS accumulation and JAG-induced cell shrinkage. The results from these experiments strongly suggest that JAG induces motor cell shrinkage through ROS accumulation and that the gene expression of *SPORK2* is indispensable in JAG-induced cell shrinkage. In addition, JAG induces shrinkage of *Samanea* motor cells independent of ABA/JA signaling.

Evidence suggests that the regulation of turgor changes in motor cells is similar to that in stomatal guard cells^49^. ABA triggers H_2_O_2_ accumulation in guard cells of *A. thaliana* through activation of the respiratory burst oxidases multigene family (RbOHs; NADPH oxidases) located on the plasma membrane^50,51^. ABA-regulated stomatal closure is impaired in the *A. thaliana Rboh D/F* (*AtrbohD/F*) mutant^52^. All of these studies indicated that ROS functions as a second messenger in ABA-induced guard cell shrinkage^35,36,42,52^. ROS as the second messenger have been shown to be involved in various intra- and intercellular signaling events. JA signaling is believed to be involved in stomatal closure^38^. The JA signaling elicitor MeJA triggers ROS accumulation in guard cells of *A. thaliana* (Fig. 2a and c)^35,36^. MeJA-activated ROS production was previously shown to be mediated by the COI1-JAZ signaling module^38^. In our study, JAG did not trigger ROS accumulation in guard cells (Fig. 2b and d), which is consistent with the non-participation of JAG in JA signaling^39^. In contrast, JAG treatment triggered ROS accumulation in extensor motor cell protoplasts, whereas MeJA treatment had no effect on ROS accumulation (Fig. 2b and d). These results suggested that JAG-triggered ROS accumulation is independent of the COI1-JAZ signaling module, consistent with a previous report^26^. DPI chloride (Fig. 4) or catalase (Fig. S3) repressed JAG-induced ROS accumulation and resulting cell shrinkage in extensor motor cell protoplasts. Considering that catalase would be effective on extracellular ROS, the results suggest that the extracellular O^2-^ produced by NADPH oxidases dismutate to H_2_O_2_, which is transported into the cytosol possibly via aquaporins.

**Figure 5:**
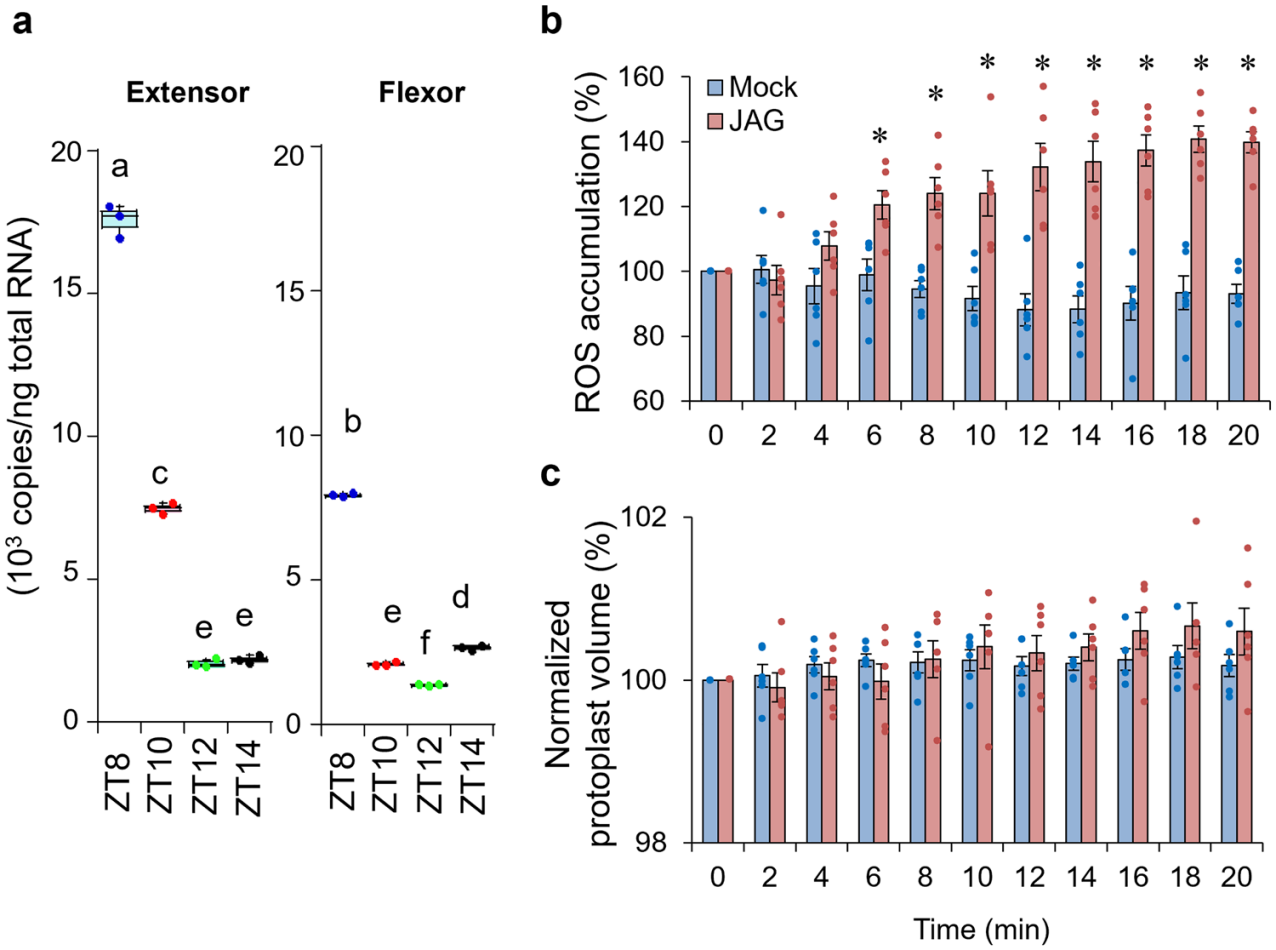
Effects of JAG on extensor protoplasts that did not express SPORK2. (**a**) The expression of *SPORK2* gene during ZT 8–14 in extensor and flexor cells. All data points are shown as dot plots (n = 3). Different letters indicate significant differences (SNK post-hoc test, *P* < 0.05). (**b**, **c**) Time course of 12-hydroxyjasmonic acid glucoside (JAG)-induced (**b**) cytosolic ROS accumulation and (**c**) cell shrinkage in tertiary extensor protoplasts of *Samanea saman* during ZT 14–18. The changes in normalized cytosolic fluorescence intensity of each protoplast were expressed by considering the negative control at each given point respectively. All data points are shown as dot plots (n = 6). Asterisks indicate significant differences (*p < 0.05; *t*-test). Three independent experiments were performed.

Recently, SPORK2 was shown to regulate leaf-movement. We found that the effect of JAG on extensor cells required SPORK2 and was time-dependent. Notably, JAG could not shrink protoplasts prepared from folded leaves between ZT 14–18 when the expression of *SPORK2* gene was at a minimum (Fig. 5), but it did induce ROS accumulation within the same time range (Fig. 5c). These results suggest that the processes of ROS accumulation and cell shrinkage are linked (by unknown mechanisms) when the leaves are open, but not when they are folded. Given that JAG-induced motor cell shrinkage was impeded by the co-addition of TEA (a blocker of K^+^ channels)^26^, our current result suggests that *SPORK2* is indispensable for JAG-triggered cell shrinkage. It has been reported that ROS accumulation and the subsequent calcium release activate outward-rectifying plant potassium channel, the GUARD CELL OUTWARD-RECTIFYING K^+^, by a calcium-dependent kinase through phosphorylation^53^. Although we did not perform calcium imaging experiments in *S. saman*, a similar phenomenon is presumed to be triggered by JAG in *S. saman*. It is also possible that ROS directly affects SPORK2, which is expressed according to circadian rhythms, to regulate its K^+^ transport activity in extensor cells, because we confirmed that SPORK2 expressed in *Xenopus* oocyte was directly activated by ROS (Fig. S4). It was reported that post-translational modification of plant K^+^ channels by ROS plays a role in the regulation of K^+^ transport. A heterologously expressed *Arabidopsis* K^+^ channel, STELAR K^+^ OUTWARD RECTIFIER, directly induces voltage-dependent activation by ROS^54^. In transmembrane 3 within the voltage sensing complex of STELAR K^+^ OUTWARD RECTIFIER, Cys-168 was responsible for its activation by ROS. Cys-168 is also present in transmembrane 3 of SPORK2, suggesting a similar activation mechanism for SPORK2 in extensor cells. Further studies will reveal the mechanism of JAG-mediated activation of SPORK2.

Based on the current finding that ROS is involved in JAG-mediated leaf-folding, the JAG-triggered shrinking of the extensor motor cell and outward-rectifying K^+^-channel SPORK2 can be linked. To elucidate the mode of action on JAG-induced cell shrinkage, the upstream components of ROS accumulation in JAG-induced cell shrinkage should be investigated in future. The target protein of JAG is the key to unmask these signaling components.

## Conclusion

In conclusion, our data demonstrate that JAG induces shrinkage of extensor motor cell protoplasts through ROS accumulation, independently of ABA/JA signaling. JAG has no effect on ROS accumulation in flexor motor cell protoplasts, but JAG can trigger ROS accumulation in the cytosol of extensor motor cell protoplasts to mediate cell shrinkage, which may trigger the folding of *Samanea* leaves. All of these results lead to the significant and novel finding that ROS is involved in JAG-induced nyctinastic leaf-folding movement.

## Experimental procedures

### Plant materials

*Arabidopsis thaliana* (Col-0) was grown in a growth chamber (LPH-240SP, Nippon medical & chemical instrument Co., Ltd., Osaka, Japan) for 4–6 weeks at 20–22 °C and 70% humidity under a 12-h-light/12-h-dark cycle [66 μmol m^-2^ s^-1^ photosynthetically active radiation (PAR)].

Seeds of *Samanea saman* were purchased from World Flower Service Co., Ltd. *S. saman* trees were grown in a growth chamber (LPH-1PH, Nippon medical & chemical instrument Co., Ltd. Osaka, Japan) under a 16/8-h light/dark regime at an intensity of 160 to 290 μmol m^-2^ s^-1^ PAR, at 25 °C ± 3 °C, and 70% relative humidity (the details of light and temperature setting for the growth chamber are: ZT 0, 20% intensity of light, 25 °C; ZT 1:00, 50% intensity of light, 27 °C; ZT 5, 100% intensity of light, 27 °C; ZT 11, 50% intensity of light, 27 °C; ZT 15, 20% intensity of light, 25 °C; ZT 16, dark, 22 °C). The study on this plant species complies with the relevant institutional, national, and international guidelines and legislation.

### Preparation of *Samanea* motor cell protoplasts for ROS detection

Tertiary pulvini protoplasts were isolated from third to fifth branch from the shoot apex of the *Samanea* trees during ZT 3–5 according to the previously reported method^15,26,55^, with modifications. Approximately 100 of the tertiary pulvini were separated into extensor (adaxial) part and flexor (abaxial) part with a sharp razor blade and placed in 1 mL of predigestion solution [Gamborg’s B-5, 0.3 M sorbitol, 50 mM MES-KOH (pH 5.5), 0.2% BSA, 8 mM CaCl_2_]. The osmotic pressure of the predigestion solution was then raised to 0.6 M sorbitol in two steps over 20 min with osmotic adjustment solution [Gamborg’s B-5, 4.0 M sorbitol, 50 mM MES-KOH (pH 5.5), 0.2% BSA, 8 mM CaCl_2_]. Tissues were then moved into a ϕ 35 mm tissue culture dish with 1.6 mL filtered enzyme solution [Gamborg’s B-5, 50 mM MES-KOH (pH 5.5), 0.4 M sorbitol, 0.2% BSA, 8 mM CaCl_2_, 3% (w/v) each of Driselase (Aska Pharmaceutical Co. Ltd., Tokyo, Japan), Macerozyme R-10, and cellulase Onozuka RS, 0.3% pectolyase Y-23 (Yakult Pharmaceutical Industry Co., Ltd., Tokyo, Japan)]. Tissues in the enzyme solution were incubated with mild shaking for 1 h at 30 °C, followed by incubation without shaking for 1 h at 30 °C. The enzyme solution was discarded and the tissues were rinsed thrice with 1 mL of recovering solution [Gamborg’s B-5, 0.35 M sorbitol, 20 mM MES-Tris (pH 5.5), 100 mM KCl, and 1 mM CaCl_2_]. The protoplasts were released in 1.6 mL recovering solution for 0.5–1 h at 30 °C and debris removed by filtration of the protoplast suspension through a 50 μm nylon mesh; this step was repeated twice. The collected protoplasts were incubated at room temperature (24 °C) for 3–4 h. Afterward, the protoplasts were concentrated on a sucrose cushion [0.57 M sucrose, 20 mM MES-Tris (pH 5.5), 10 mM KCl, 1 mM CaCl_2_] by centrifugation at 60 × *g* for 5 min and subsequently purified on sucrose gradient: protoplasts were suspended with 0.8 mL ~ 80% sucrose cushion in a 2 mL Eppendorf tube, then 0.5 mL mix solution [sucrose cushion:wash solution = 4:3; wash solution: 0.57 M sorbitol, 20 mM MES-Tris (pH 5.5), 10 mM KCl, 1 mM CaCl_2_] was layered on top of the protoplast suspension and 0.5 mL wash solution was layered in the upper part. The gradient was centrifuged at 130 × *g* for 10 min. The purified protoplasts were collected at the interphase between wash solution and mixed solutions. The yield of protoplasts was 1.5 to 3 × 10^5^.

### Measurement of ROS for motor cell protoplasts of *S. saman* using CLSM

The prepared protoplasts (10,000 cells/mL) in 130 µL wash solution were sealed in a glass-bottom Petri dish (φ 35 mm × 12 mm), coated with 200 µL of H_2_O, and incubated overnight at 24 ± 1 °C in dark. Then, the protoplasts were added to 5 µM H_2_DCF-DA (Sigma-Aldrich Co., Ltd., MO, USA) and incubated for 45 min to stabilize their initial fluorescence intensity. Thereafter, the protoplasts were imaged by CLSM (LSM 700, Carl Zeiss, Oberkochen, Germany) at 2-min intervals for 20 min after treatment with 100 µM JAG^26^ dissolved in 0.1% DMSO, H_2_O_2_ (FUJIFILM Wako Pure Chemical Industries Co., Osaka, Japan) at the indicated concentration, or mock solution (0.01% ethanol or DMSO for DPI); untreated protoplasts acted as the blank control. When used, 12.5 µM DPI (Sigma-Aldrich Co., Ltd., MO, USA) dissolved in DMSO or 100 U/mL catalase (Sigma Co., Ltd.) was added 30 min before treatment with the above compounds. Intercellular fluorescence was excited using 488 nm light emitted by a solid-state diode laser at 0.5% with a Plan-Apochromat 40 × /1.3 oil immersion objective and other settings as follows: emission 495–628 nm, master gain 500–650, pinhole 0.9 µm, 8-bit, frame 1024 × 1024 pixel, zoom 1.0, pixel dwell time 1.58 µs/pixel and line average of 4. Autofluorescence was negligible in this emission range using these settings. Zen 2012 Black Edition software (Carl Zeiss, Oberkochen, Germany) was used for image analysis. Photobleaching and dye leakage from the intercellular to adjacent areas was too low to detect under these conditions. Dye leakage from the cytosol to the vacuole was assessed by comparing the levels of vacuole fluorescence at the beginning and end of each experiment. The round protoplasts were selected in which vacuole fluorescence intensity was less than twice as strong as that of background solution. However, the protoplast was discarded if dye leakage from the cytosol to the vacuole had increased the intensity of vacuole fluorescence up to 200%. ROS accumulation was calculated based on the fluorescence intensity of H_2_DCF-DA. The ROS accumulation of H_2_DCF-loaded protoplasts induced by blue light and the dark conditions was recorded with untreated protoplasts as blank. ROS accumulation was estimated with the following equation:

$$ {\text{ROS accumulation }}\left( \% \right) \, = \left( {\frac{{Fc_{n} }}{{Fu_{ave. of n} }} \div \frac{{Fc_{0} }}{{Fu_{ave. of 0} }}} \right) \times 100 $$

(Fc_n_ = the fluorescence intensity of a protoplast treated with chemicals at nth minute. n = 0, 2, 4, … 20. Fu_ave.of n_ = average of the fluorescence intensity of untreated protoplasts at nth minute. Fc_0_ = the fluorescence intensity of a protoplast treated with chemicals at 0 min. Fu_ave.of 0_ = average of the fluorescence intensity of untreated protoplasts at 0 min). Pixel intensities of fluorescence at each given time were collected as the average intensity of three points that were away from the chloroplasts and vacuoles in each cell. Data were collected from two experiments in parallel on the same day.

### Measurement of cell shrinkage of motor cell protoplasts of *S. saman* using CLSM

The protoplasts selected for measuring the cytosol fluorescence intensity were used for measuring the cell shrinkage. First, the intensity of the ROS signal was adjusted into similar-level contrast in the same protoplasts at the denoted times. Then, a red circle was made to fit the edge (critical surface of ROS signal) of the round part of the protoplast. The area of the red circle was calculated based on the average area of two independent fitting processes, and the changes in normalized protoplast volume was calculated.

### Quantitative RT-PCR analysis of *SPORK2*

To analyze time-course gene expression profiles of *SPORK2* in *Samanea* tertiary pulvini, excised extensor and flexor motor cells were sampled every 2 h from ZT 8 to ZT 14. Total RNA was isolated using the RNeasy Plant Mini Kit (QIAGEN, Hilden, Germany), and cDNA was synthesized using ReverTra Ace (TOYOBO, Osaka, Japan) with oligo(dT)_20_ primers. Quantitative PCR was performed on a StepOnePlus Real-Time PCR System (Thermo Fisher Scientific, CA, USA) with KAPA SYBR Fast qPCR Kit (KAPA Biosystems, MA, USA). The following primers were used; forward, 5′-TGCTGGTAAAATCACCAATACC-3′, reverse, 5′- GCCGTGATAAATTATCACAC-3′.

### The statistical analysis

All data are presented as means ± SE except denotation. The values followed by different letters are statistically different according to analysis of variance followed by SNK post hoc test. Besides, the significance of differences between data sets was assessed by Student’s t-test. Differences were considered significant for *P* value < 0.05.

## Supplementary Information

Supplementary Information.

## Data Availability

The datasets generated and/or analyzed during the current study are reported in the references cited or available from the corresponding author, Minoru Ueda, upon request.
