# Supplementary material for: 12-Hydroxyjasmonic acid glucoside causes leaf-folding of Samanea saman through ROS accumulation
Source: Sci Rep. 2022 May 4;12:7232. doi: 10.1038/s41598-022-11414-2 (PMC9068819; doi:10.1038/s41598-022-11414-2)
Supplement: Supplementary file 1 — Supplementary Information. [file 41598_2022_11414_MOESM1_ESM.docx]

***Supporting Information for*:**

**12-Hydroxyjasmonic acid glucoside causes leaf-folding of *Samanea saman* through the ROS accumulation**

**Authors:**

Gangqiang Yang,^1,4^ Yasuhiro Ishimaru,^1^ Shunji Hoshino,^2^ Yuki Muraoka,^1^ Nobuyuki Uozumi,^3^ and Minoru Ueda^1,2,*^

**Affiliations:**

^1^Department of Chemistry, Graduate School of Science, Tohoku University, Sendai 980-8578, Japan

^2^Department of mechanism and Chemical Life Sciences, Graduate School of Life Sciences, Tohoku University, Sendai 980-8578, Japan

^3^Graduate School of Engineering, Tohoku University, 6-6-07, Aobayama, Aoba-ku, Sendai 980-8579, Japan.

^4^Current affiliation: School of Pharmacy, Collaborative Innovation Center of Advanced Drug Delivery System and Biotech Drugs in Universities of Shandong, Key Laboratory of Molecular Pharmacology and Drug Evaluation, Ministry of Education, Yantai University, Yantai, 264005, China

E-mail: [minoru.ueda.d2@tohoku.ac.jp](mailto:minoru.ueda.d2@tohoku.ac.jp)

**Contents**

**Figure S1–S7**

**Supporting Methods**


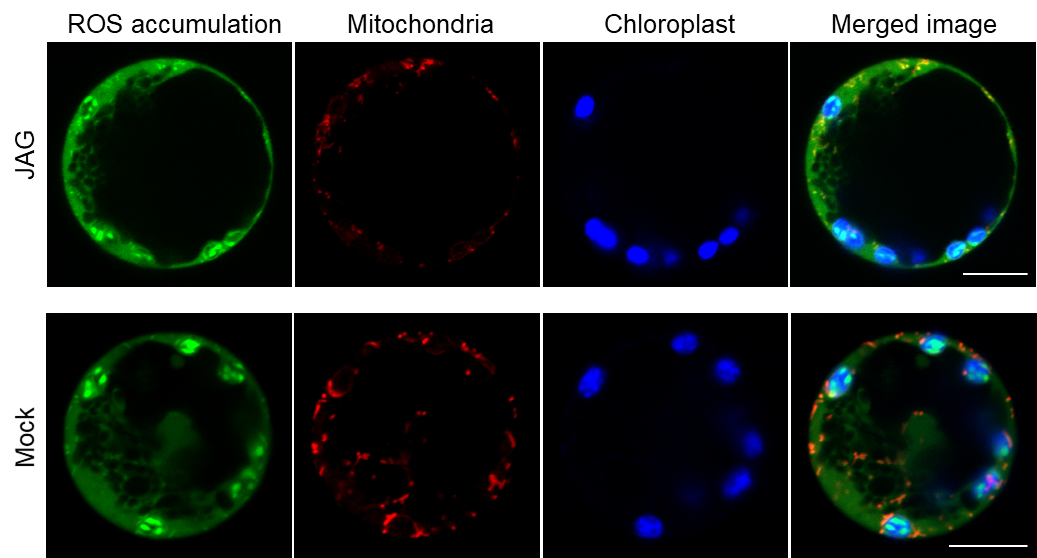


**Figure S1**. **Distribution of JAG-induced ROS accumulation** **in extensor motor cell protoplasts**

Tertiary extensor motor cell protoplasts of *S. saman* were double-stained with H_2_DCFDA (green) and MitoTracker Red CMXRos (red) for 45 min before treating with 100 µM JAG or Mock. Images were observed by CLSM after 15 min of the treatment. Chloroplast autofluorescence (blue) was excited at 488 nm and visualized above 640 nm. This experiment was repeated three times with similar results. Bar = 10 µm.


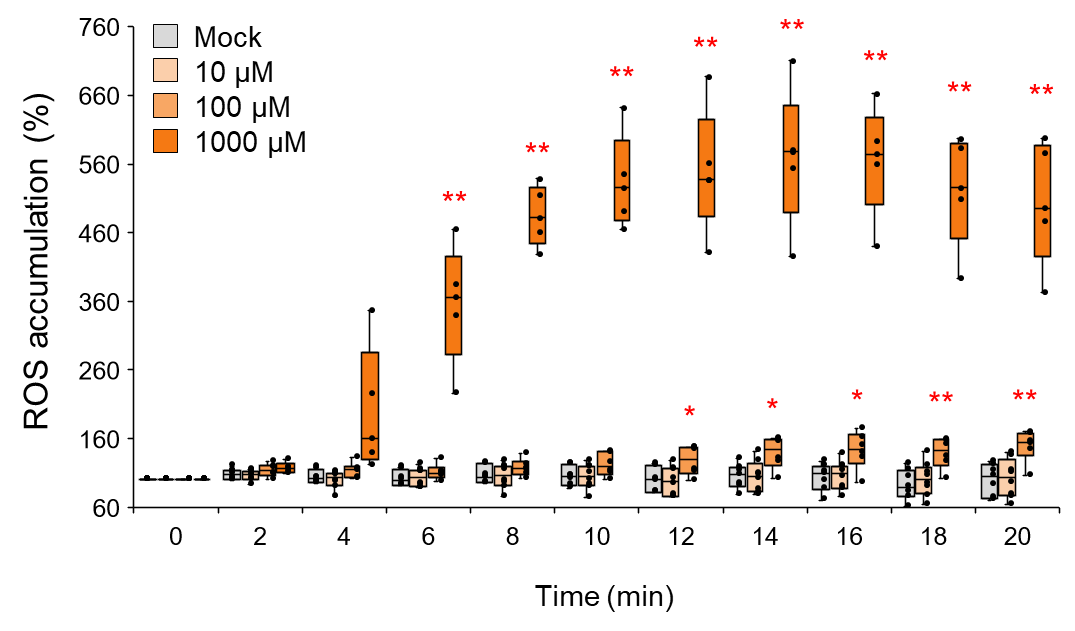


**Figure S2**. **Effect of different concentrations of H_2_O_2_ on ROS accumulation in tertiary extensor protoplasts of *S. saman***

Protoplasts were treated with 10,100, 1000 µM H_2_O_2_, or mock. Box plots represent time course of H_2_O_2_ induced cytosolic ROS accumulation. All data points are shown as dot plots (n = 5-8). Asterisks indicate significant differences (*p < 0.05, **p < 0.01; t test). Three independent experiments were performed.

**
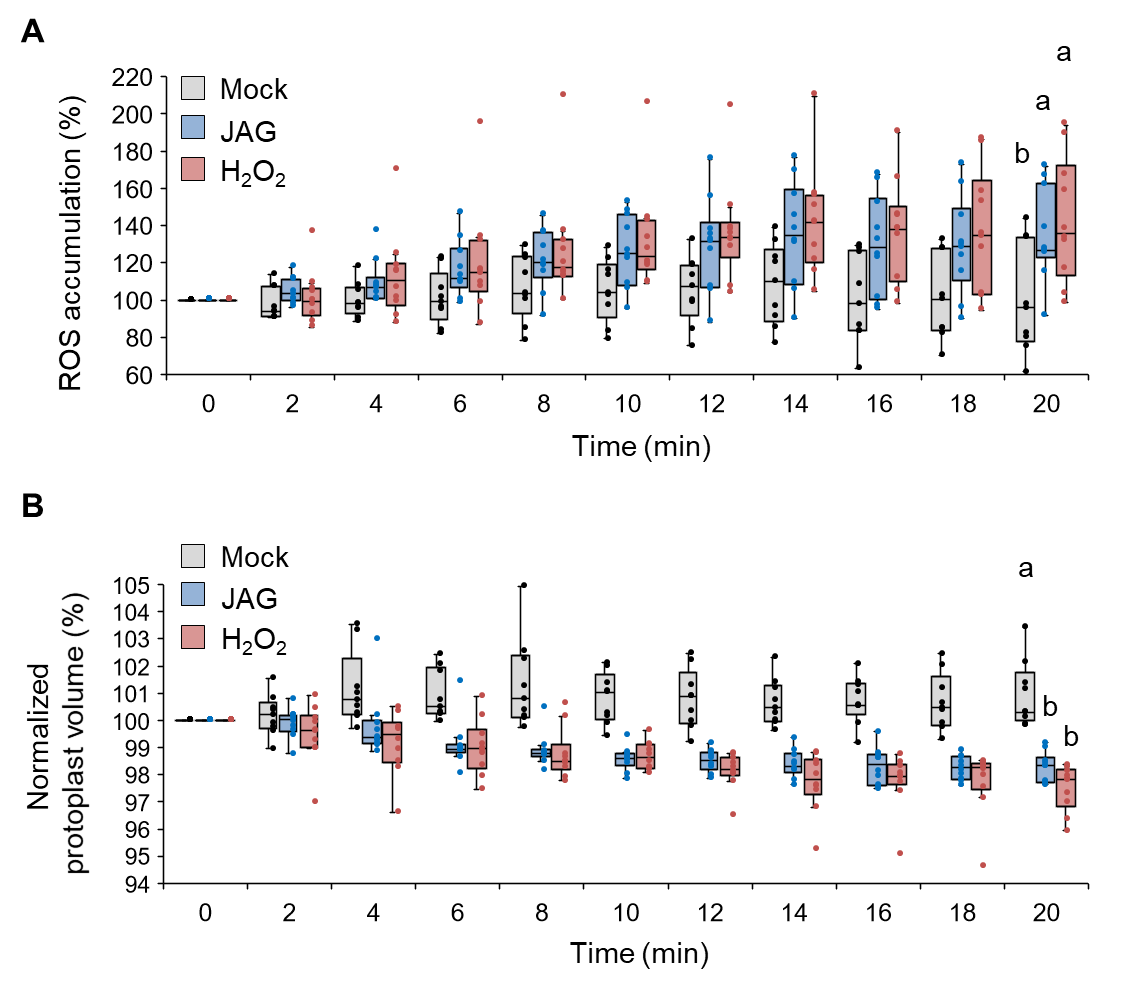
**

**Figure S3. H_2_O_2_ induced cell shrinkage in tertiary extensor protoplast of *S. saman***

Box plots represent **t**ime course of JAG and H_2_O_2_ induced cytosolic ROS accumulation (**A**) and cell shrinkage (**B**) in tertiary extensor protoplasts of *S. saman* during ZT 6-11. All data points are shown as dot plots (n = 9-10). Protoplasts were treated with 100 µM JAG (blue squares), 100 µM H_2_O_2_ (red squares) or mock (gray squares). Different letters indicate significant differences (SNK post-hoc test, P < 0.05). All experiments were conducted during ZT 6-11. Three independent experiments were performed.

**
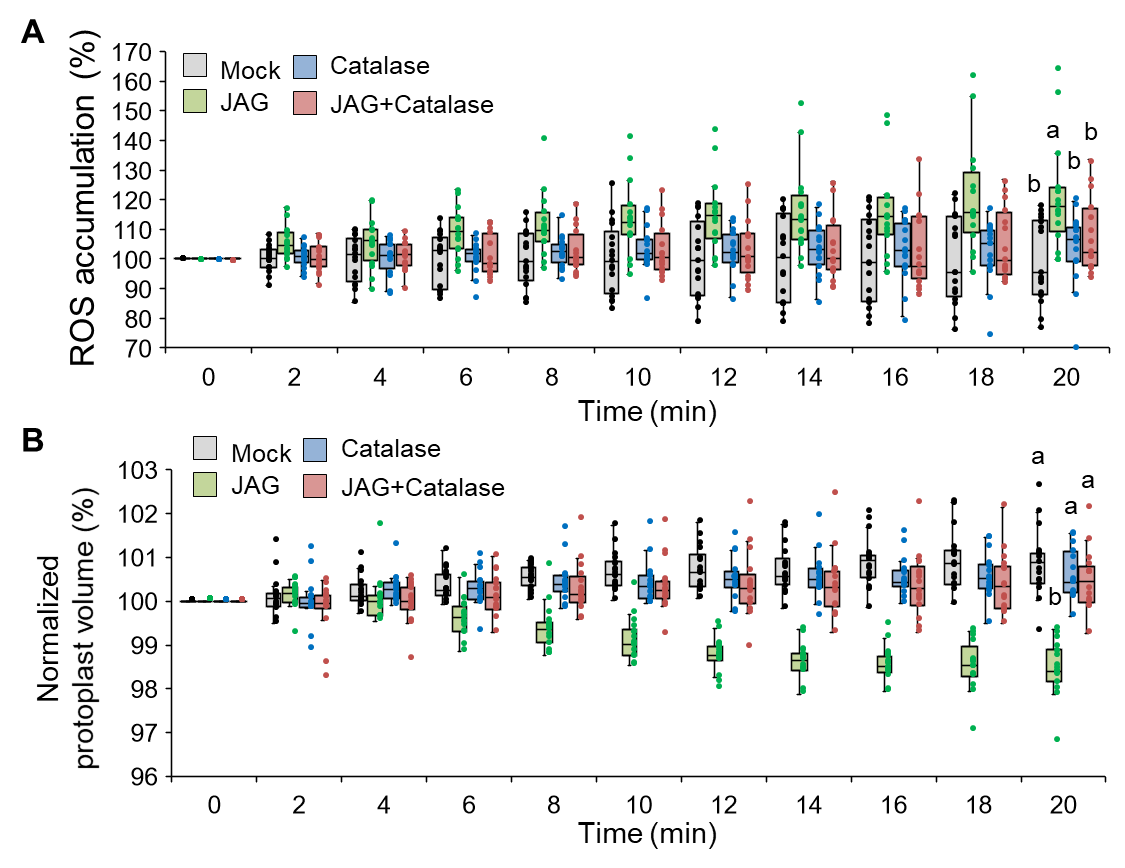
**

**Figure S4**. **Effect of catalase on JAG induced ROS accumulation in the tertiary extensor cells of *S. saman***

(**A**) and (**B**) Effect of catalase on the JAG-induced cytosolic ROS accumulation (**A**) and cell shrinkage (**B**) in the tertiary extensor protoplasts of *S. saman* during ZT 6-11. Protoplasts were preincubated for 30 min with 100 U/ml catalase before 100 µM JAG was added. All data points are shown as dot plots. Different letters indicate significant differences (SNK post-hoc test, P < 0.05). Four independent experiments were performed.

**
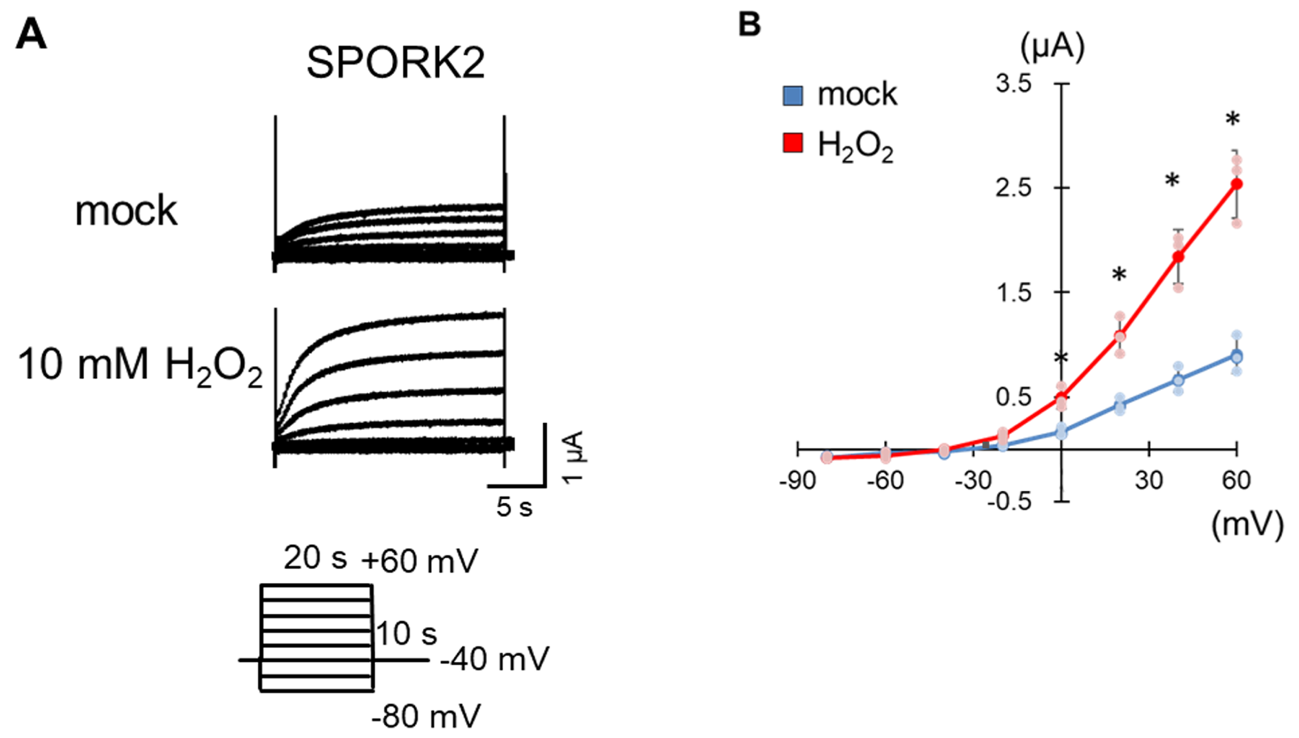

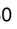
**

**Figure S5. Activation of K^+^-transport activity of SPORK2 by H_2_O_2_**

**(A)** Current traces for SPORK2 before (upper) and after (lower) adding 10 mM H_2_O_2_ in extracellular solution.

**(B)** Currents for SPORK2 before and after adding 10 mM H_2_O_2_ plotted against each clamped voltage. Data are the mean ± SD (n = 3). Asterisks indicate significant differences (*p < 0.05; t test). These experiments were repeated three times with similar results.


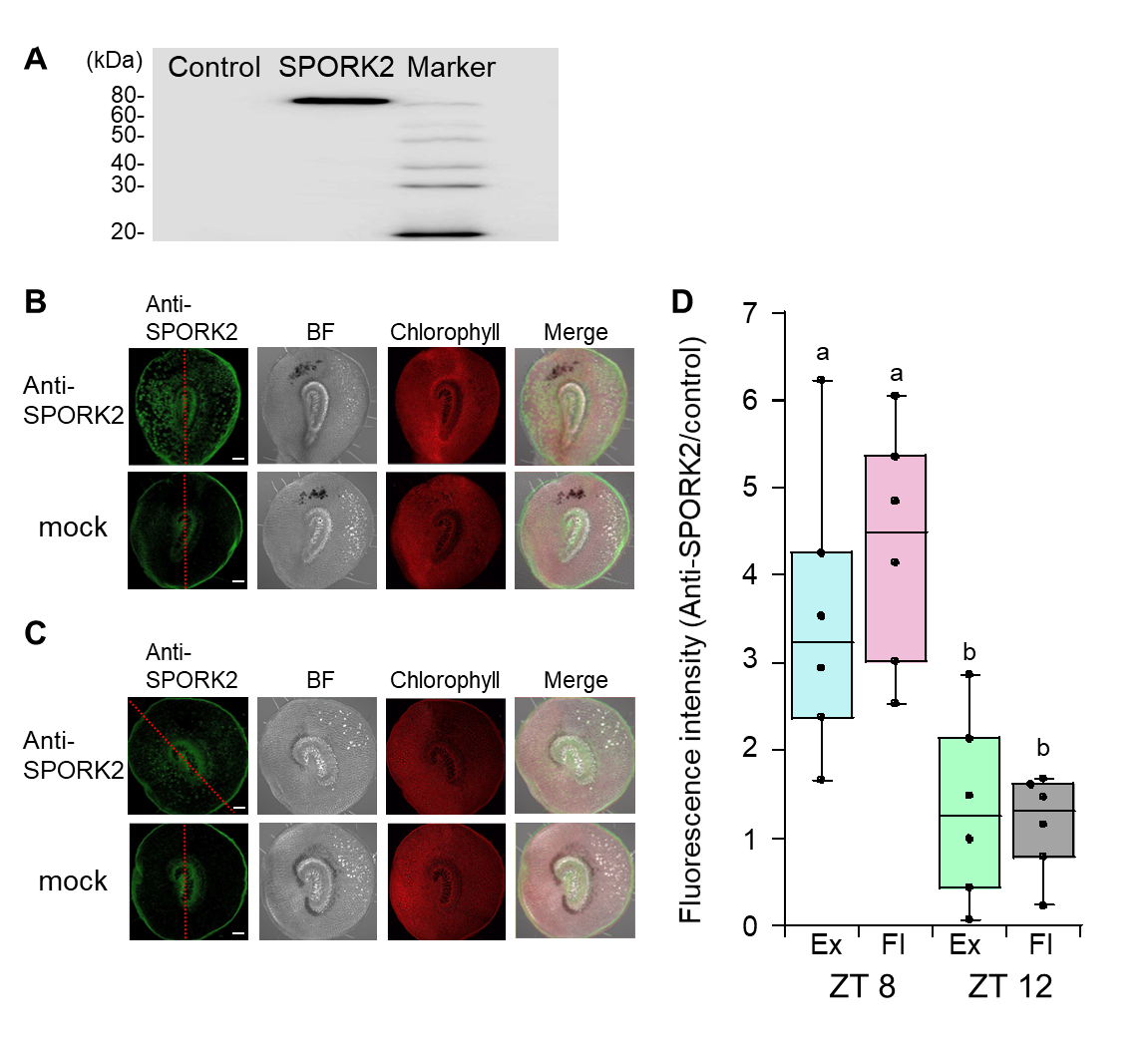


**Figure S6. Expression of SPORK2 protein in *Samanea* tertiary pulvinus**

(A) Western blot analysis of SPORK2 expressed in *Xenopus* oocytes by using anti-SPORK2 antibody. *Xenopus* oocytes expressing SPORK2 (middle lane) or not (control, left lane) were used.

(B) and (C) Fluorescence images of SPORK2 protein expression visualized by anti-SPORK2 antibody in *Samanea* tertiary pulvini. The red dotted lines separate the adaxial sides containing extensor motor cells (left side) and the abaxial side containing flexor motor cells (right side). Chlorophyll autofluorescence (red) was excited at 488 nm and emission >640 nm was recorded. Tertiary pulvini were sampled at ZT 8 (B) or ZT 12 (C). Bar = 250 μm. These experiments were repeated at least six times with similar results.

**(D)** Ratios of fluorescence intensity immunostained for SPORK2 relative to that for mock in the tertiary pulvini. All data points are shown as dot plots (n = 6). Different letters indicate significant differences (SNK post-hoc test, P < 0.05).


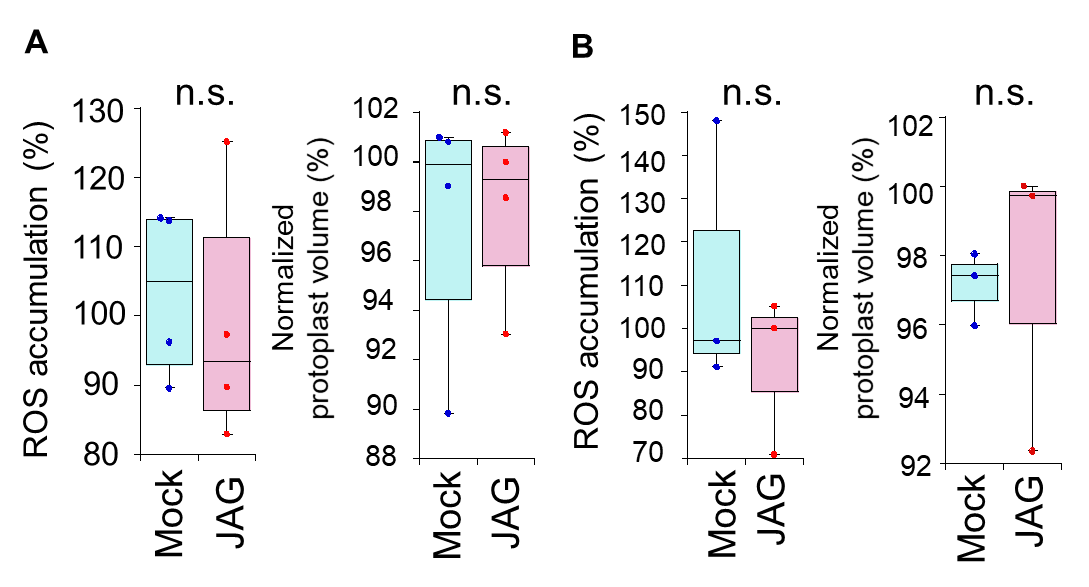


**Figure S7. Flexor motor cells protoplasts do not accumulate ROS and shrink after JAG treatment in ZT 14-18 and ZT 0-4**

**(A)** Box plots represent JAG-induced cytosolic ROS accumulation and cell shrinkage in tertiary flexor protoplasts of *S. saman* after 20 min adding 100 μM JAG in ZT 14-18. Protoplasts were loaded with H_2_DCFFA for 45 min before adding JAG. All data points are shown as dot plots (n = 4).

**(B)** Box plots represent JAG induced cytosolic ROS accumulation and cell shrinkage in tertiary flexor protoplasts of *S. saman* at 20 min after treatment in ZT 0-4. Protoplasts were loaded with H_2_DCFFA for 45 min before adding 100 μM JAG. All data points are shown as dot plots (n = 3). Significant differences were not found by t test (P < 0.05) both in (A) and (B). Three independent experiments were performed.

**Supporting Methods**

**Measurement of ROS for guard cells of *A. thaliana***

ROS accumulation in guard cells was evaluated using 2’, 7’-dichlorodihydrofluorescein diacetate (H_2_DCF-DA).[^30^](#_ENREF_30)^,^[^31^](#_ENREF_31) Epidermal tissues were incubated in medium containing 5 mM KCl, 50 µM CaCl_2_, and 10 mM MES-Tris (pH 6.15) in the light at 22 °C. After 3 h, 50 µM H_2_DCF-DA was added to the sample. Epidermal tissues were incubated for 30 min at room temperature in the dark, collected with a 100-µm nylon mesh, and twice washed with distilled deionized water. The dye-loaded tissues were treated with 10 µM MeJA (FUJIFILM Wako Pure Chemical Co., Osaka, Japan), 10 µM ABA (Tokyo Chemical Industry Co., Tokyo, Japan), 100 µM JAG or 0.01% ethanol (mock) in the dark at room temperature. After 20 min, the fluorescence of the guard cells was imaged using a fluorescence microscope (IX-71, Olympus, Tokyo, Japan) with U-MNIBA3 filter (excitation wavelength 470-495 nm; absorption wanvelength 510-550 nm; dichroic mirror wavelength, 505 nm, Olympus, Tokyo, Japan). Prolonged exposure of the H_2_DCF-loaded guard cells to the original excitation beam led to a transient increase in ROS and then photobleaching.[^30^](#_ENREF_30) The excitation beam exposure was reduced by double neutral density filters (ND6 filter and ND12 filter, Olympus, Tokyo, Japan) and limited to 10 seconds. The image was recorded under magnification ≥×160 using a digital camera (DP 72, Olympus, Tokyo, Japan). ImageJ 1.46r (NIH, Bethesda, MD, USA) was used for image analysis. Average background fluorescence of H_2_DCF around each pair of guard cells was subtracted. Each datum was randomly obtained from 60 pairs of guard cells with fluorescence emission in seven pieces of epidermal tissues and no more than 10 pairs of guard cells were used from the same tissue. Three independent experiments were performed.

**Measurement of ROS for tertiary extensor protoplasts of *S. saman***

ROS accumulation in the tertiary extensor protoplasts of *S. saman* was evaluated as for the guard cells of *A. thaliana*, with minor modifications. The prepared protoplasts in 130 µL wash solution (10,000 cells) were incubated in a black plate with clear bottom (Corning Inc., Corning, NY, USA) overnight at 24 ± 1 ºC in dark. Then, 5 µM H_2_DCF-DA was added and the protoplasts were incubated for 30 min. Finally, the protoplasts were treated by the above compounds for 15 min during ZT 6-11. Fluorescence intensities were measured as described above; each datum was randomly obtained from 100 protoplasts with fluorescence emission in triple parallel experiments. Three independent experiments were performed.

**Immunostaining of *Samanea* tertiary pulvini**

The pulvini were immersed in PEMT solution (50 mM PIPES (pH 7.2), 2 mM EGTA, 2 mM MgSO_4_, 0.05% Triton-X-100) containing 1.25% [w/v] paraformaldehyde and 0.5% [v/v] glutaraldehyde on ice followed by agitation for 40 min for immobilization. After immobilization, samples were washed with PEMT solution for 10 min three times. The pulvini were then embedded in 4% agarose and sliced at a thickness of 50 µm using microslicer (SUPER MICROSLICER ZERO 1, DOSAKA EM Co., Ltd., Kyoto, Japan). Afterwards, samples were treated for 25 min at 30°C in PEMT solution containing 0.05% [w/v] pectolyase Y-23 (Kikkoman Co., Chiba, Japan) and 0.4 M mannitol. Then washed with PEM solution (50 mM PIPES (pH 7.1), 2 mM EGTA, 2 mM MgSO_4_) for 5 min three times followed by washing with PEM solution containing 0.5% tween for 10 min. The samples were treated with phosphate buffered saline (PBS) solution containing 1 mg/mL NaBH_4_ for 10 min to quench aldehyde-derived autofluorescence then washed twice for 5 min with incubation buffer (PBS solution containing 50 mM glycine). The samples were treated with incubation buffer containing 1% BSA for 30 min for blocking, followed by antibody treatment. Rabbit anti-SPORK2 antibody (produced by BioGate, 1~~,~~00-fold dilution), which was raised against a synthetic peptide corresponding to a SPORK2 N-terminal amino-acid sequence (D35-S50), was treated at 4 °C overnight. After washing with incubation buffer (10 min, three times), sheep anti-rabbit IgG-DyLightTM 488 (ROCKLAND) was treated at 37 °C for 3 hours. Finally, the samples washed with PBS (10 minutes, six times), and preparations were made for observation. CLSM (LSM 700, Carl Zeiss, Oberkochen, Germany) was used to detect SPORK2 protein: laser at 488 nm, laser power at 1, pinhole at 71.5, and gain at 700-800. A bandpass filter was used to pass only the 490-555 nm wavelength. The objective lens was a 2X or 10X magnifying lens for detection. Fluorescence intensities in the abaxial sides or the adaxial sides were measured using Image J 1.52v software (<http://imagej.net/Welcome>).

**Western blot analysis using *Xenopus* oocytes**

Oocytes expressing SPORK2 were homogenized in extraction buffer (50 mM Tris-HCl (pH 8.0), 800 mM sucrose, 5 mM EDTA, 5 mM DTT and complete^®^). The supernatant was then collected by centrifugation at 1,000 g for 5 min at 4 °C. The collected supernatant was further centrifuged three times at 15,000 g for 1 h at 4 °C. The supernatant was collected and incubated for 10 min at 60 °C. Then the samples were loaded on SDS-PAGE and analyzed with western blotting. SPORK2 were detected using rabbit anti-SPORK2 antibody (BioGate, 1,000-fold dilution in PBS containing 0.1% Tween 20 and 2% Immunoblot Blocking Reagent (Millipore)) and goat anti-rabbit IgG-HRP antibody (sc-2303, Santa Cruz biotechnology, 10,000-fold dilution in PBS containing 0.1% tween 20 and 2% Immunoblot Blocking Reagent).

**Observation of morphology and caspase-like activities in *Samanea* extensor motor cell protoplasts**

Protoplasts were treated as described for the measurement of ROS by CLSM. To analyze caspase-like activities, the CaspACE FITC-VAD-fmk in situ marker kit (Promega, UK) was used. Protoplasts were loaded with 1 µM FITC-VAD-fmk for 30 min, then treated with 100 µM JAG, 100 µM H_2_O_2_ or a 10 min-pulse of heat shock at 55 ºC.([Swidzinski et al., 2002](#_ENREF_59); [Vacca et al., 2004](#_ENREF_66); [de Pinto et al., 2013](#_ENREF_8)) Photomicrographs were taken by CLSM at 20 or 120 min after treatment. Caspase-like activities were visualized and appeared as fluorescent green in cells. The change in the morphology of the protoplasts was visualized in transmission pictures. Three independent experiments were performed.

**Viability of *Samanea* extensor motor cell protoplasts**

A suspension of protoplasts was prepared at a concentration of 5 × 10^5^ cells/mL in wash solution and incubated overnight at 24 ± 1 ºC in the dark. A solution of 100 µM JAG or 100 µM H_2_O_2_ in wash solution (2 µL) was added to 30 µL of the protoplast suspension. After incubation at 24 ± 1 ºC in the dark for 20 or 120 min and staining with 16 µL of 0.4% (w/v) trypan blue solution (Wako Pure Chemical Industries, Ltd.) living protoplasts were counted under a microscope. Protoplasts were treated with a 10 min-pulse of heat shock at 55 ºC ([Swidzinski et al., 2002](#_ENREF_59); [Vacca et al., 2004](#_ENREF_66); [de Pinto et al., 2013](#_ENREF_8)) using a Dry Thermo Unit (DTU-1B; Taitec, Saitama, Japan) as positive control. The basal viability of the overnight incubated protoplasts was calculated from the ratio of living cells to total cells. Then, the viability for each treatment was calculated from the ratio of the number of living protoplasts under a given treatment to that under basal conditions. Three independent experiments were performed.

**Oocyte preparation, cRNA synthesis and microinjection**

Ovary lobes were surgically removed from female *Xenopus* laevis (Hamamatsu Seibutsu Kyozai, Shizuoka, Japan) and washed with Barth’s solution w/o Ca^2+^ (88 mM NaCl, 1 mM KCl, 0.82 mM MgSO_4_, 2.4 mM NaHCO_3_, 5 mM Tris/HCl (pH 7.4)). Ovaries were then gently shaken with Barth’s solution w/o Ca^2+^ containing 0.05% (w/v) type IA collagenase at 20 ºC for 90 min. After washing with Barth’s solution (88 mM NaCl, 1 mM KCl, 0.33 mM Ca(NO_3_)_2_, 0.41 mM CaCl_2_, 0.82 mM MgSO_4_, 2.4 mM NaHCO_3_, 5 mM Tris/HCl (pH 7.4)), oocytes were gently shaken in Barth’s solution at 20 ºC for 10 min. After washing again, the oocytes were shaken in Barth’s buffer w/o Ca^2+^ at 20 ºC for 10 min. Defolliculated oocytes were stored in Barth’s solution containing 50 mg/mL gentamicin at 18 ºC. cRNA synthesis and oocyte microinjection were performed as described previously [^58^](#_ENREF_58).

**Electrophysiological experiments to evaluate K^+^ channels using *Xenopus* oocytes**

Whole-oocyte current was recorded using a two-electrode voltage clamp method with an AxoClamp 2B (Molecular Devices, CA, USA) as previously described (Kato *et al.,* 2001). Data analysis was performed using an Axon Digidata 1550 Low-Noise Data Acquisition System (Molecular Devices, CA, USA). To measure K^+^ current, oocytes were perfused in bath containing a solution of 108 mM NaCl, 12 mM KCl, 1 mM MgCl_2­­_, 1 mM CaCl_2_, and 10 mM HEPES/NaOH (pH 7.4) at room temperature (23 ± 2 ℃). Step pulses were elicited from -80 mV to +60 mV in 20 mV increments, starting from a holding potential of -40 mV. Current values were measured at the end of 20 s pulses at each membrane potential. When necessary, H_2_O_2_ was directly added to the bath solution just before measurements. All experiments were performed 3-4 d after cRNA injection.

**Statistical analysis**

All data are presented as mean ± SE except were indicated. Different letters indicate significant differences (SNK post-hoc test, P < 0.05). The significance of differences between data sets was assessed by Student’s t-test (P < 0.05).
